# Supplementary material for: Cu single-atom embedded g-C3N4 nanosheets rehabilitate multidrug-resistant bacteria infected diabetic wounds via photoswitchable cascade reaction
Source: Nat Commun. 2025 Oct 16;16:9198. doi: 10.1038/s41467-025-64242-z (PMC12533187; doi:10.1038/s41467-025-64242-z)
Supplement: Supplementary file 1 — Supplementary Information [file 41467_2025_64242_MOESM1_ESM.pdf]

Supplementary Information

Cu Single-Atom Embedded g-C_3N_4 nanosheets Rehabilitate Multidrug-Resistant Bacteria Infected Diabetic Wounds via Photoswitchable Cascade Reaction

Xichen Sun[1,2†], Pengqi Zhu[3,4†], Liuyan Tang[1,2], Pengfei Wang[1,2], Ningning Li[1,2], Qing Wang[1,2], Yan-Ru Lou[5], Yuezhou Zhang[1,2], and Peng Li[1,2,6]**

[1]State Key Laboratory of Flexible Electronics (LoFE) & Institute of Flexible Electronics (IFE), Frontiers Science Center for Flexible Electronics (FSCFE), Northwestern Polytechnical University, 127 West Youyi Road, Xi'an, 710072, China

[2]Key laboratory of Flexible Electronics of Zhejiang Province, Ningbo Institute of Northwestern Polytechnical University, 218 Qingyi Road, Ningbo, 315103, China

[3]Shanxi Bethune Hospital, Shanxi Academy of Medical Sciences, Tongji Shanxi Hospital, Third Hospital of Shanxi Medical University, 99 Longcheng Street, Taiyuan, 030032, China

[4]Tongji Hospital, Tongji Medical College, Huazhong University of Science and Technology, Wuhan, 430030, China

[5]Faculty of Pharmacy, University of Helsinki, P.O. Box 56, FI-00014 Helsinki, Finland

[6]Research and Development Institute of Northwestern Polytechnical University, Shenzhen, 518057, China

† These authors contributed equally to this work.

E-mail: iamyzzhang@nwpu.edu.cn; iampli@nwpu.edu.cn

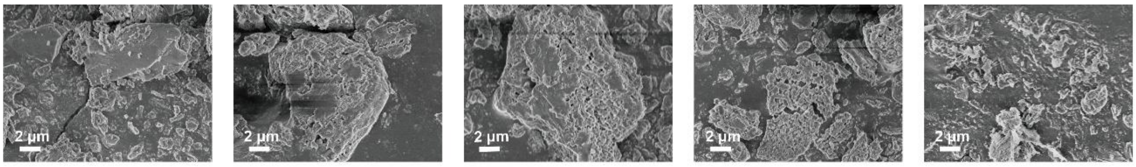

CN_{550} CN_{600} CN_{650} CN_{700} CN_{750}

Figure S1. SEM of CN treated at 550 °C, 600 °C, 650 °C, 700 °C and 750 °C.

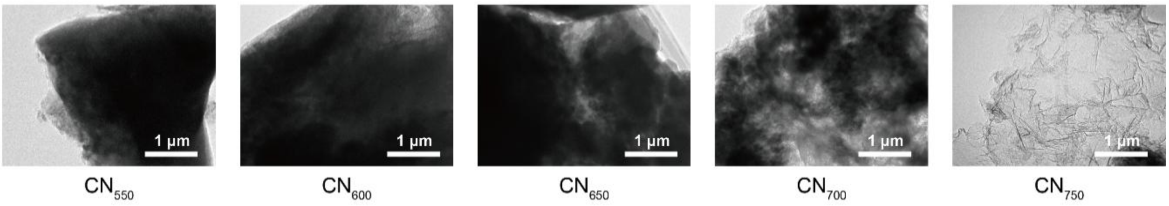

Figure S2. TEM of CN treated at 550 °C, 600 °C, 650 °C, 700 °C and 750 °C.

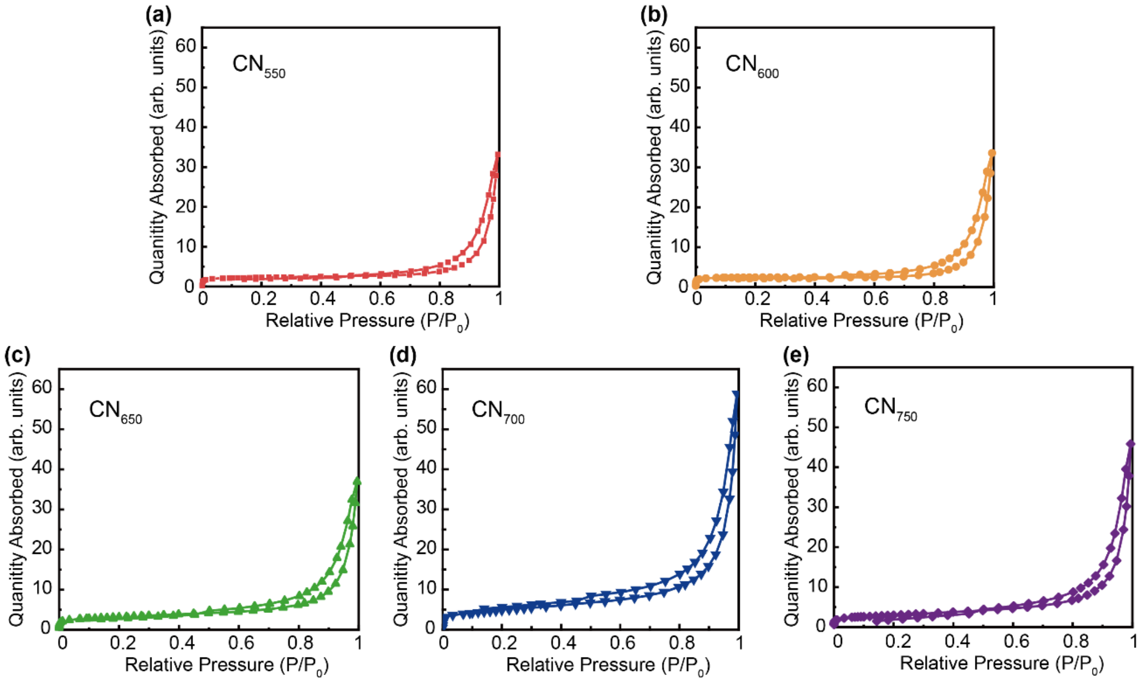

Figure S3. BET of CN treated at 550 °C, 600 °C, 650 °C, 700 °C and 750 °C.

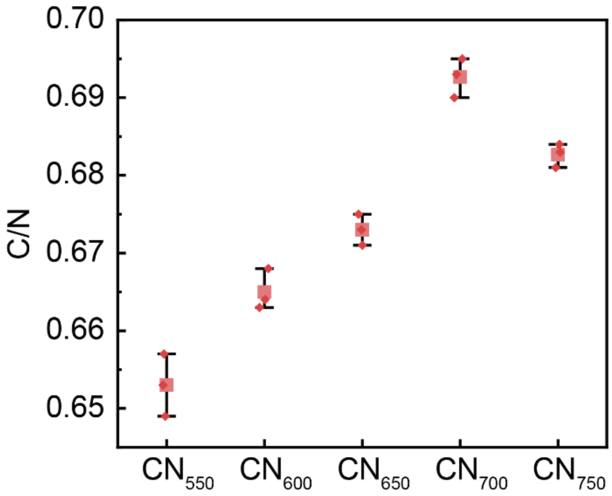

Figure S4. The C/N mass ratio characterized by elemental analysis of CN_{550}, CN_{600}, CN_{650}, CN_{700} and CN_{750}.

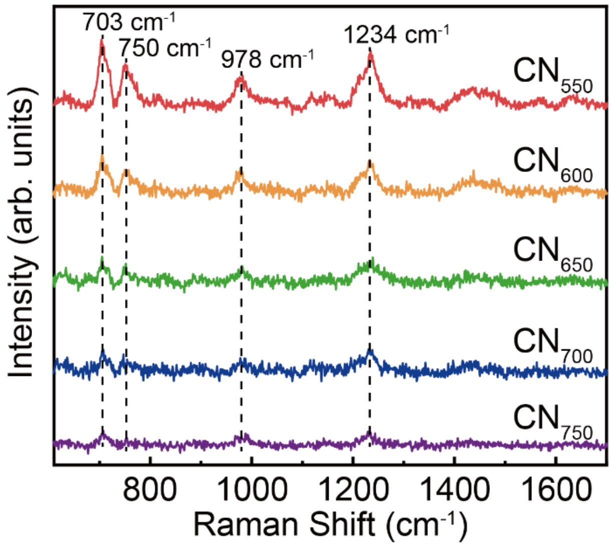

Figure S5. The Raman spectra of CN_{550}, CN_{600}, CN_{650}, CN_{700} and CN_{750} excited with a NIR laser, 785 nm.

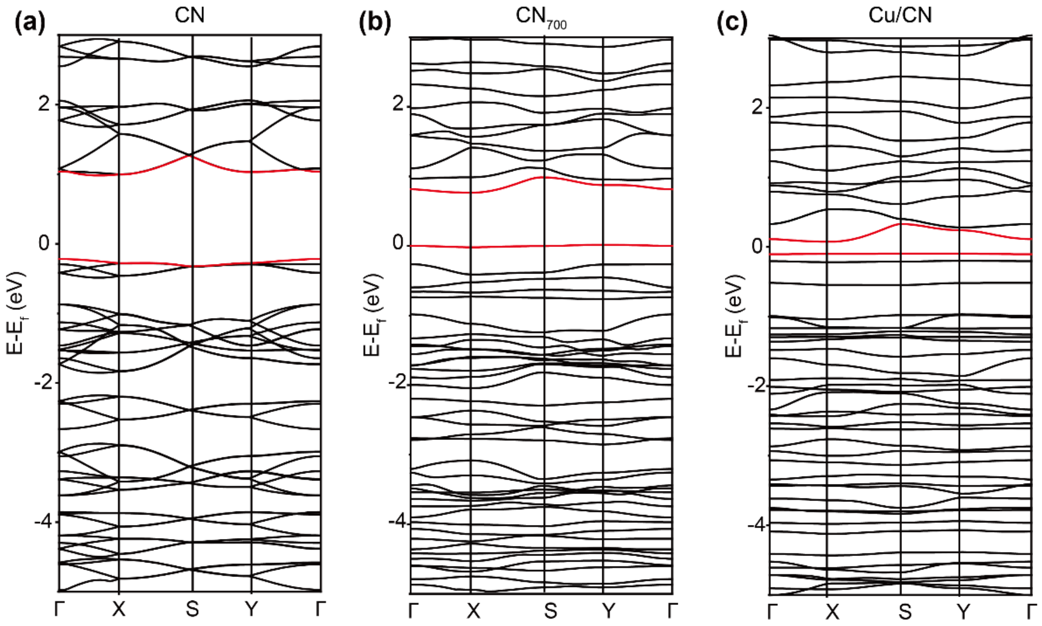

Figure S6. PBE of (a) CN, (b) CN_{700}, and (c) Cu/CN.

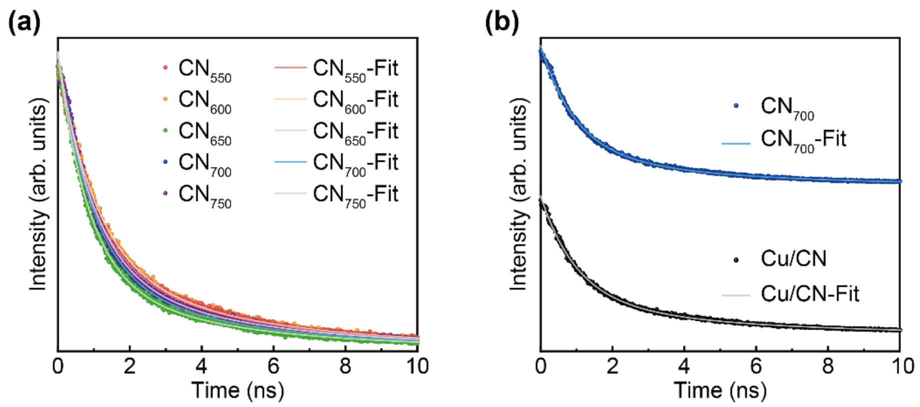

Figure S7. (a) The TRPL spectra of CN_{550}, CN_{600}, CN_{650}, CN_{700} and CN_{750}. (b) The TRPL spectra of CN_{700} and Cu/CN.

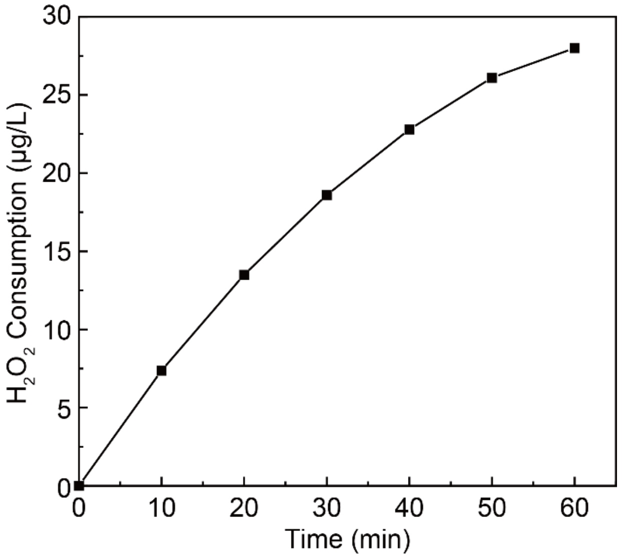

Figure S8. Kinetics of H_2O_2 consumption with Cu/CN.

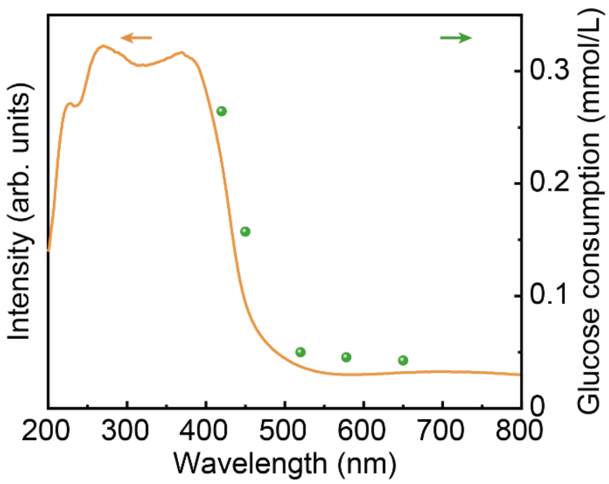

Figure S9. Glucose consumption activity of Cu/CN under different wavelength (0.2 W/cm^2).

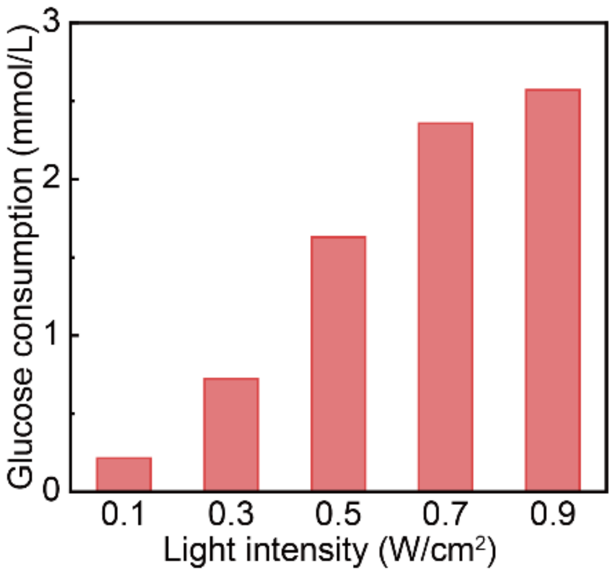

Figure S10. Glucose consumption activity of Cu/CN under different intensities of Xe light ($\lambda > 420$ nm).

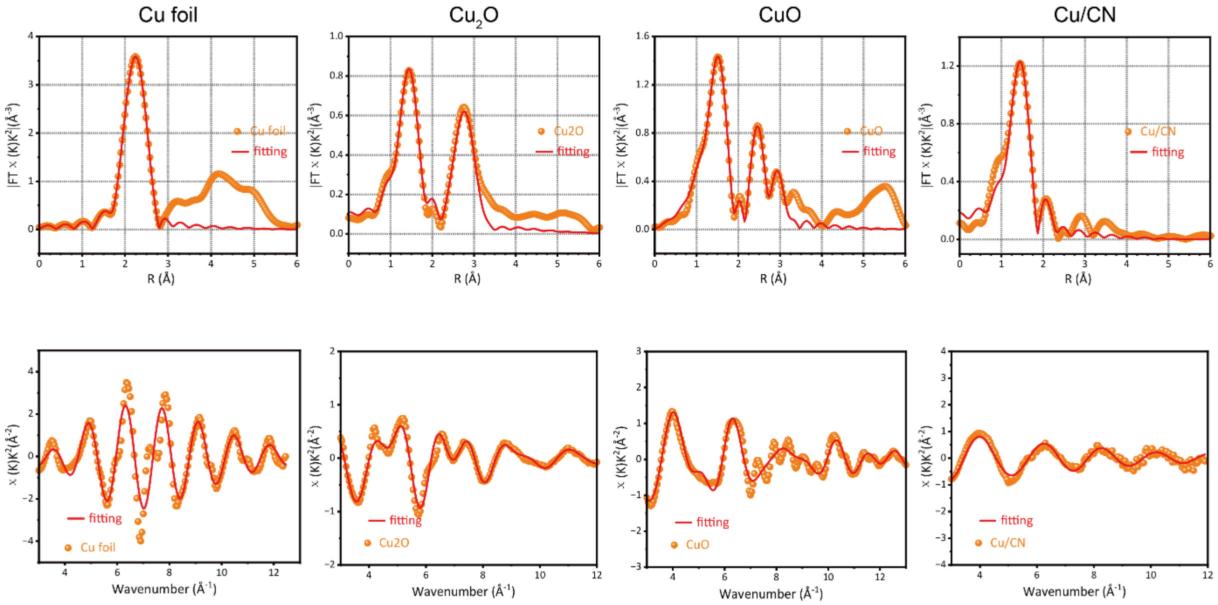

Figure S11. EXAFS R-space and K-space fitting curve of Cu foil, Cu_2O, CuO, and Cu/CN.

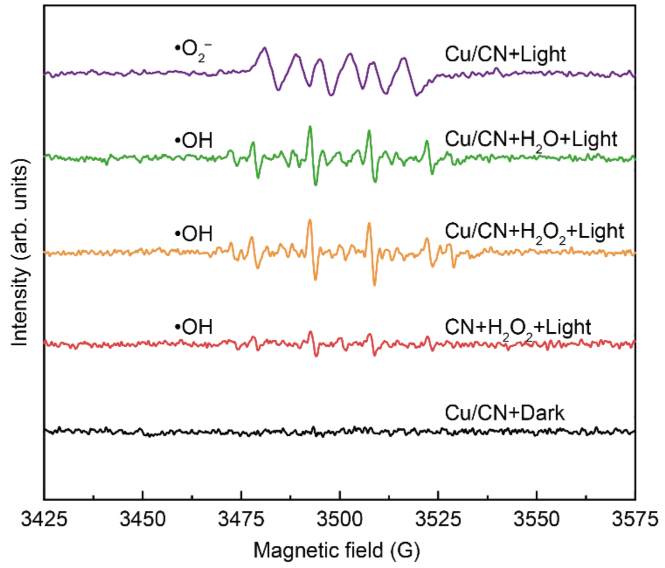

Figure S12. •OH, and •O_2^- EPR spectra of CN_{700} and Cu/CN.

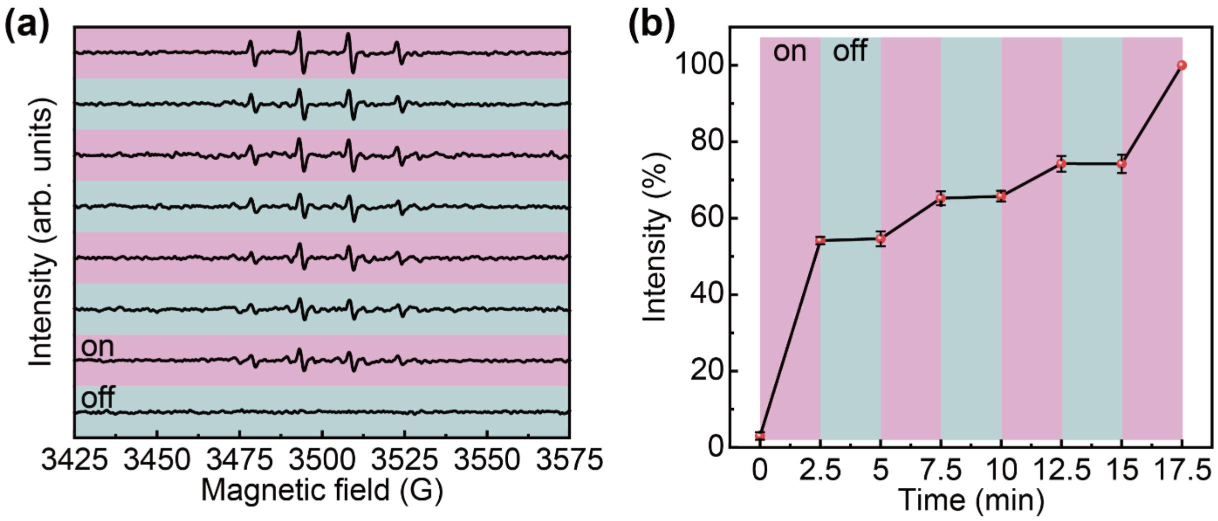

Figure S13. (a) In situ EPR detection of •OH produced by Cu/CN in aqueous glucose solution under light-dark cycles. (b) Semi-quantitative statistical analysis was performed according to the peak height of EPR in (a).

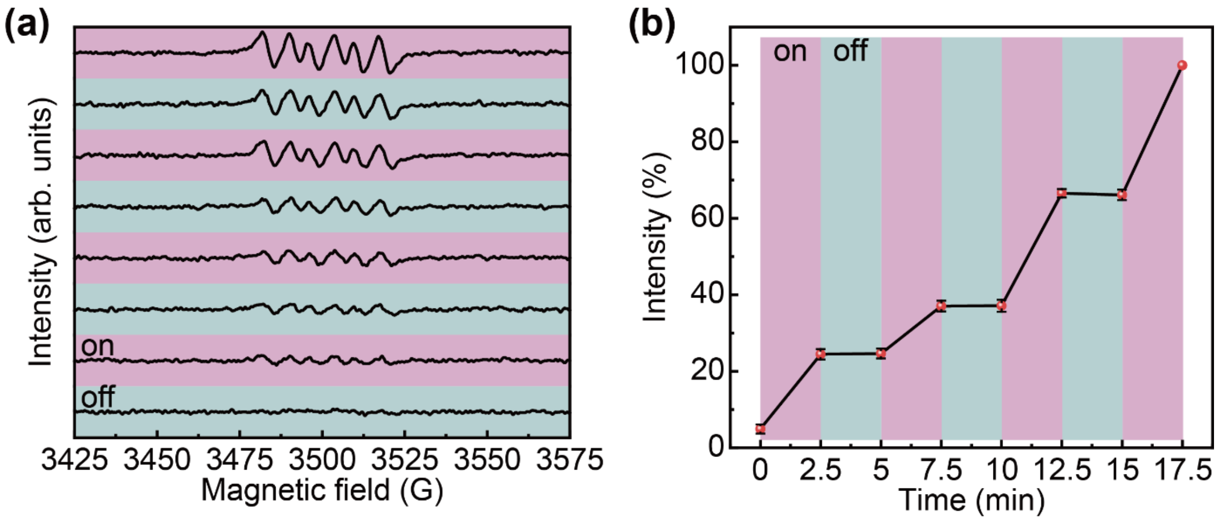

Figure S14. (a) In situ EPR detection of $\bullet O_2^-$ produced by Cu/CN in methanol solution under light-dark cycles. (b) Semi-quantitative statistical analysis was performed according to the peak height of EPR in (a).

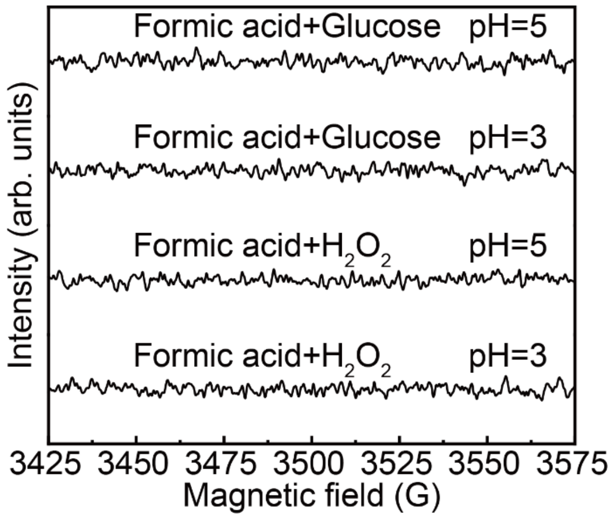

Figure S15. EPR spectroscopy of ROS produced in the dark by Cu/CN in glucose solution at different pH (pH=5 and 3) adjusted by HCOOH.

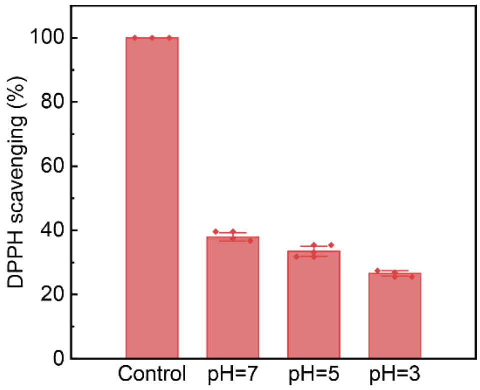

Figure S16. ROS-scavenging efficiency of Cu/CN at different pH determined by DPPH method.

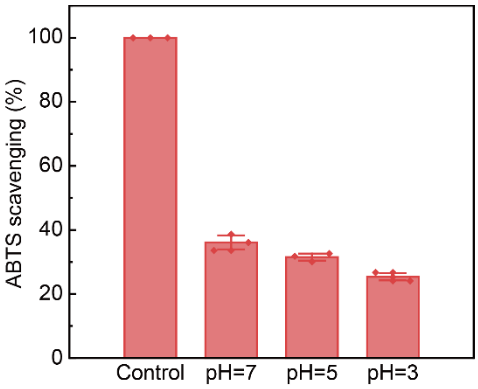

Figure S17. ROS-scavenging efficiency of Cu/CN at different pH determined by ABTS method.

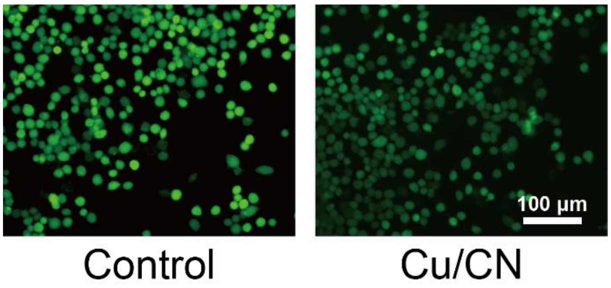

Figure S18. Intracellular ROS-scavenging performance of Cu/CN.

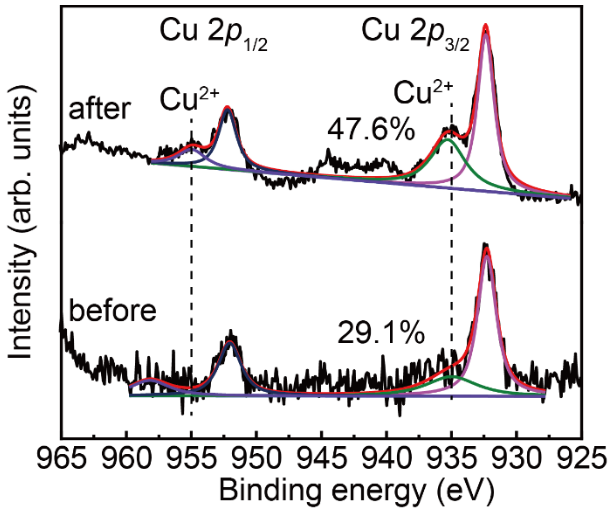

Figure S19. Cu 2*p* XPS spectra of Cu/CN before and after scavenge ROS during dark reaction.

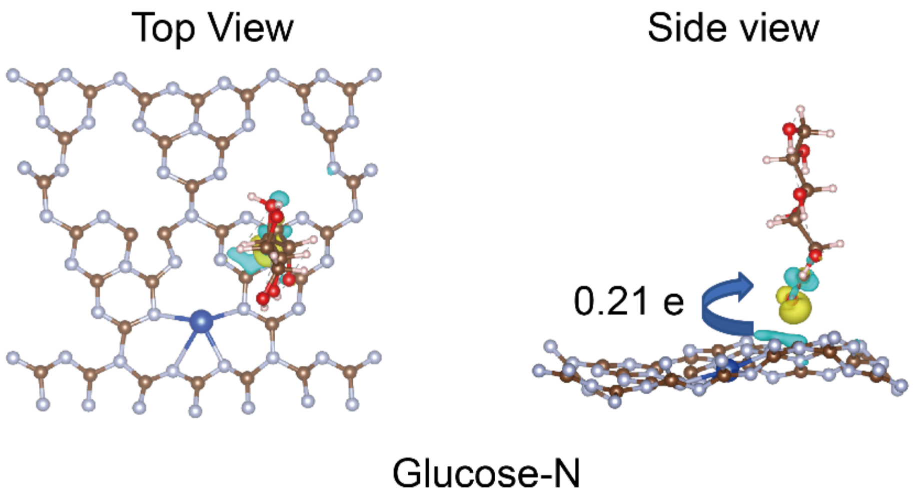

Figure S20. Top and side views of the Charge Density Difference Analysis for the glucose-N. The yellow and the cyan areas represent charge accumulation and depletion, respectively.

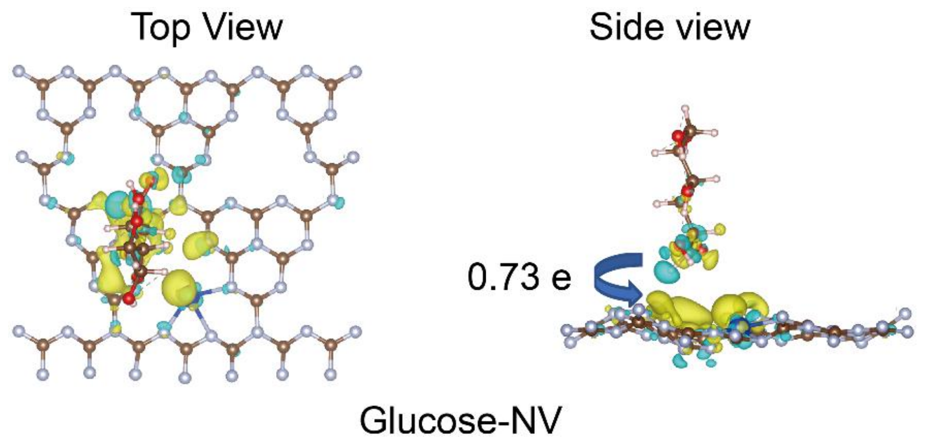

Figure S21. Top and side views of the Charge Density Difference Analysis for the glucose-NV. The yellow and the cyan areas represent charge accumulation and depletion, respectively.

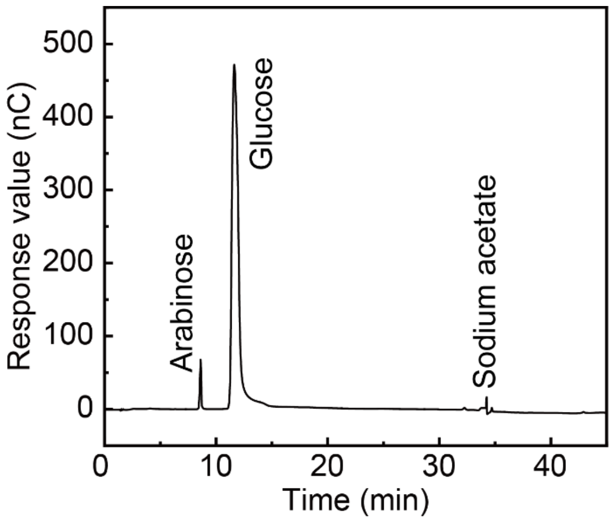

Figure S22. HPLC results of the glucose consumption.

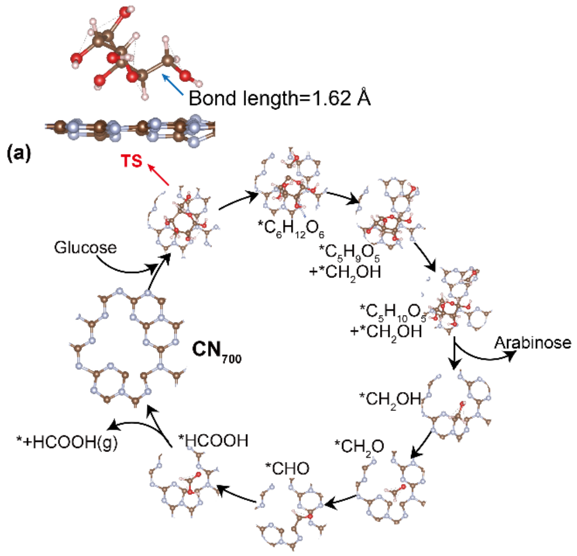

Figure S23. Proposed reaction mechanism of glucose oxidation on CN_{700} (a) and Cu/CN (b).

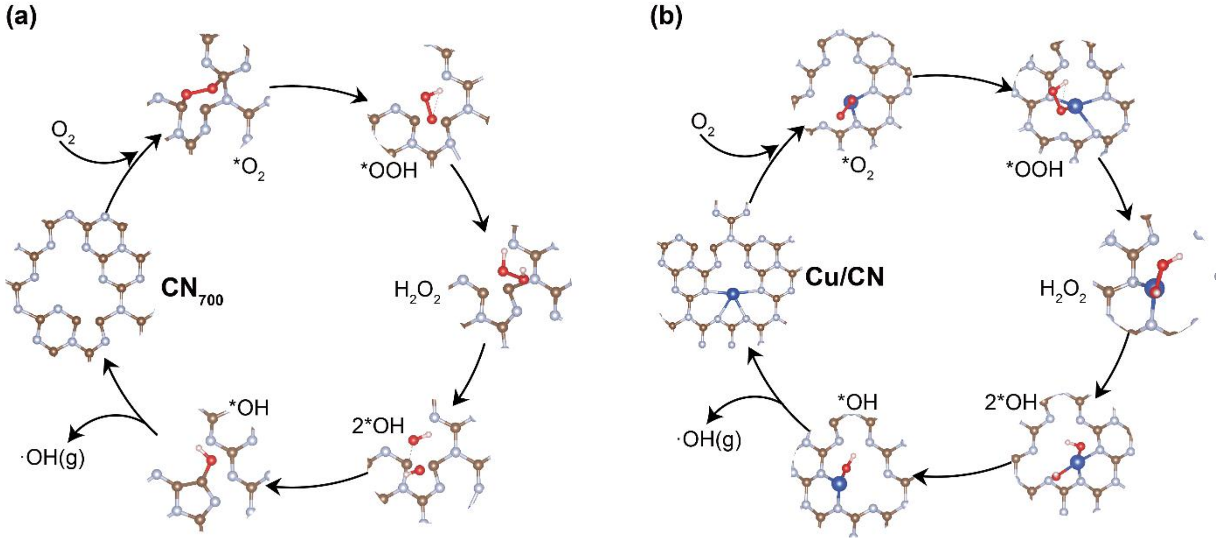

Figure S24. Proposed reaction mechanism of generation and conversion of H_2O_2 on CN_{700} (a) and Cu/CN (b).

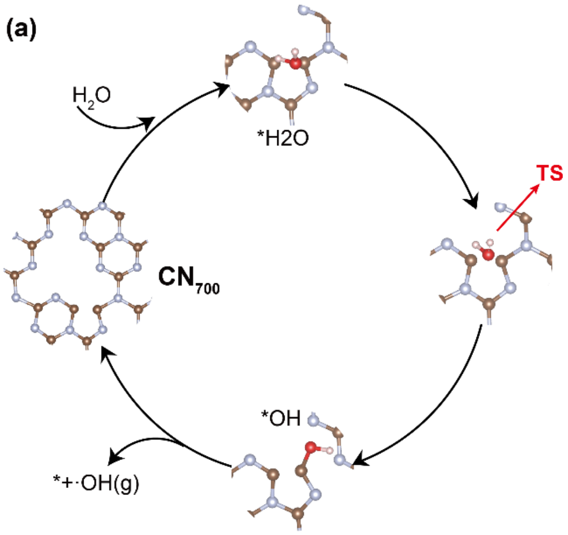

Figure S25. Proposed reaction mechanism of water splitting on CN_{700} (a) and Cu/CN (b).

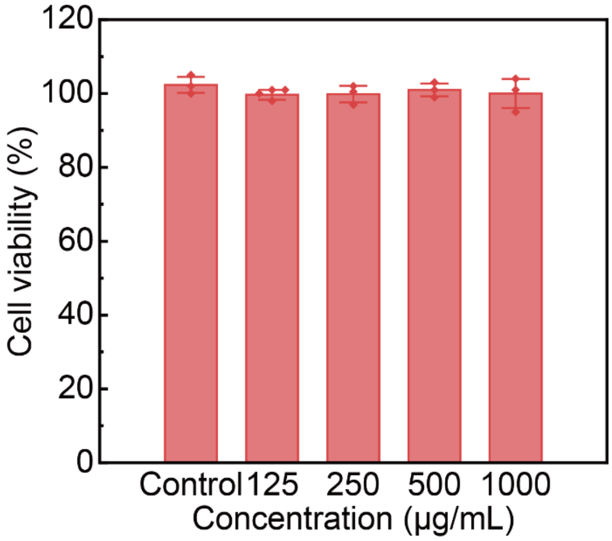

Figure S26. Cell viability of 3T3 cells incubated with Cu/CN at different concentrations.

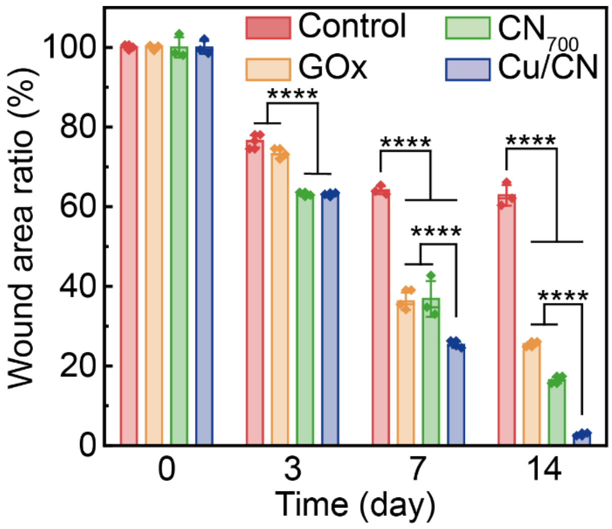

Figure S27. Quantitative analysis of wound area in different groups treated with light (λ>420 nm). *P* values were calculated by the one-way ANOVA method. ****(p<0.0001), Cu/CN vs. control for day 14, Data were presented as mean value ± SD. The experiments were repeated three times independently with similar results.

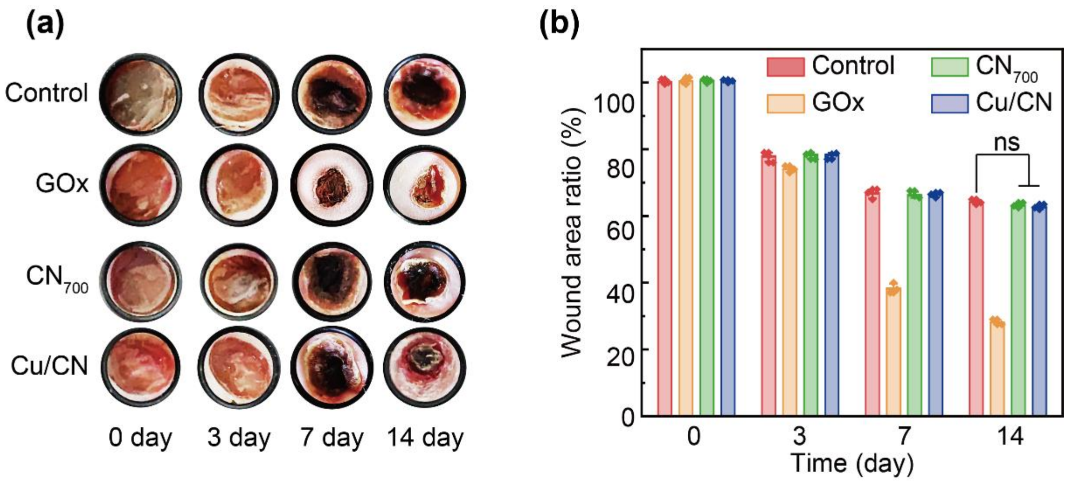

Figure S28. (a) Photographs of the wound closure procedure treated without light. (b) Assessment of the wound area ratios. *P* values were calculated by the one-way ANOVA method. (b) ns ($p > 0.05$): $p = 0.1533$, Cu/CN vs. control for day 14. Data were presented as mean value ± SD. The experiments were repeated three times independently with similar results.

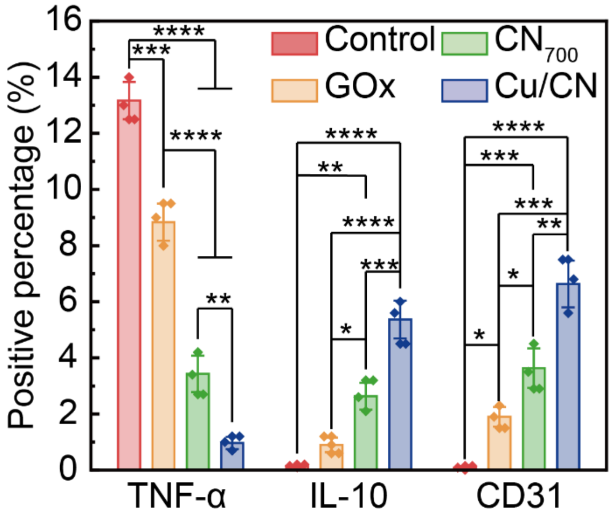

Figure S29. Fluorescence quantitative analysis of TNF-α, IL-10, and CD31 from different treatment groups on day 14. *P* values were calculated by the one-way ANOVA method. ****($p<0.0001$): Cu/CN vs. control for TNF-α, IL-10, and CD31. Data were presented as mean value ± SD. The experiments for (b, d) were repeated three times independently with similar results.

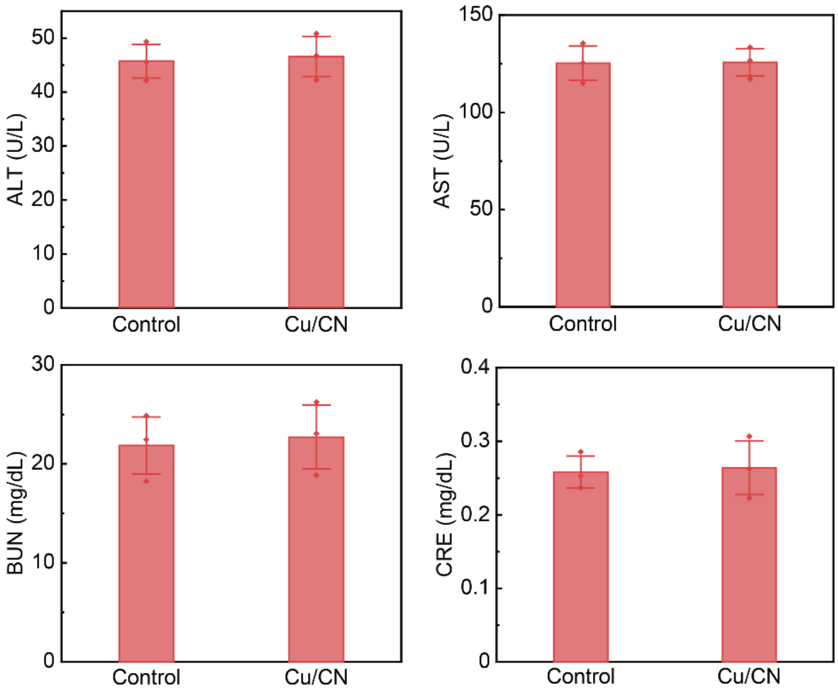

Figure S30. Serum biochemical markers (ALT, AST, BUN, CRE) (n = 3 independent experiments) were quantified post-treatment (day 14) across control and Cu/CN groups.

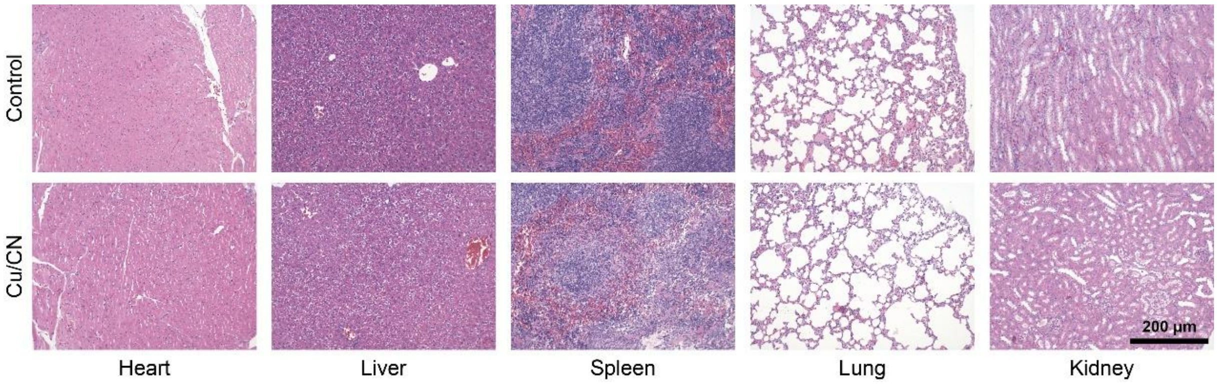

Figure S31. H&E staining of major organs of mice after different treatments.

Table S1. The specific surface area and pore size of CN

Entry	sample	SBET ($m^2\ g^{-1}$)	Pore Volume ($cm^3\ g^{-1}$)
1	CN_{550}	7.2909	0.007877
2	CN_{600}	7.3677	0.007493
3	CN_{650}	10.9141	0.012754
4	CN_{700}	16.7939	0.022491
5	CN_{750}	10.3465	0.015052

Table S2. Charge carrier density (N_d) of CN_{550}, CN_{600}, CN_{650}, CN_{700}, and CN_{750}.

Entry	Photocatalyst	$d(1/C^2/dV_s)$	N_d (cm^{-3})
1	CN_{550}	4.8821×10^7	1.8522×10^{21}
2	CN_{600}	3.9056×10^7	2.3153×10^{21}
3	CN_{650}	3.8556×10^7	2.3453×10^{21}
4	CN_{700}	2.8251×10^7	3.2008×10^{21}
5	CN_{750}	4.4798×10^7	2.0185×10^{21}

Table S3. Potentials of CN_{550}, CN_{600}, CN_{650}, CN_{700}, and CN_{750}.

Entry	Photocatalyst	$E_{Ag/AgCl}$ (V)	E_{RHE} (V)	E_{NHE} (V)
1	CN_{550}	-0.5698	0.0284	-0.1716
2	CN_{600}	-0.5869	0.0113	-0.1887
3	CN_{650}	-0.6615	-0.0633	-0.2633
4	CN_{700}	-0.7696	-0.1714	-0.3714
5	CN_{750}	-0.5874	0.0108	-0.1892

TableS4. EXAFS fitting parameters at the Cu K-edge for various samples

Sample	Shell	CN^a	R(Å)^b	σ^2(Å^2)^c	ΔE_0(eV)^d	R factor
Cu foil	Cu-Cu	12.0*	2.53±0.01	0.0082	5.5	0.0005
Cu_2O	Cu-O	1.5±0.2	1.84±0.01	0.0025	6.6	
	Cu-Cu	12.0*	3.04±0.01	0.0225	8.8	0.0167
CuO	Cu-O	4.0*	1.95±0.01	0.0044	-1.3	
	Cu-Cu	4.0*	2.91±0.01	0.0059		0.0083
	Cu-Cu	6.0*	3.10±0.01	0.0090	3.7	
Cu/CN	Cu-N	4.3±0.3	1.98±0.01	0.0037	-2.8	0.0103

^{a}*CN*, coordination number; ^{b}*R*, distance between absorber and backscatter atoms; $^c\sigma^2$, Debye-Waller factor to account for both thermal and structural disorders; $^d\Delta E_0$, inner potential correction; *R* factor indicates the goodness of the fit. S_0^2 was fixed to 0.91. A reasonable range of EXAFS fitting parameters: $0.700 < S_0^2 < 1.000$; $CN > 0$; $\sigma^2 > 0$ Å^2; $|\Delta E_0| < 15$ eV; R factor < 0.02.

Table S5. Average lifetimes of photogenerated charge carriers in CN_{550}, CN_{600}, CN_{650}, CN_{700}, CN_{750} and Cu/CN.

Model	ExpDec3					
Equation	$y = A_1*exp(-x/\tau_1) + A_2*exp(-x/\tau_2) + A_3*exp(-x/\tau_3) + y_0$					
Catalyst	CN_{550}	CN_{600}	CN_{650}	CN_{700}	CN_{750}	Cu/CN
y_0	0.00041	-0.0008	-0.00194	-0.00389	-0.00214	-0.00568
A_1	0.63514	0.69787	0.75869	0.76205	0.74971	0.73335
τ_1	0.92878	1.03703	0.8402	0.95102	0.96847	0.97981
A_2	0.32827	0.29858	0.25565	0.2564	0.27101	0.27147
τ_2	3.54217	4.04169	3.55719	3.99582	4.00381	4.128
A_3	0.04603	0.03621	0.02814	0.02748	0.03248	0.03151
τ_3	15.85249	21.58852	24.99563	31.86752	25.74288	35.21762
τ_{ave}	6.53972 ns	8.29735 ns	9.48876 ns	12.45349 ns	10.03749 ns	15.06038 ns
