# Supplementary material for: Cu single-atom embedded g-C3N4 nanosheets rehabilitate multidrug-resistant bacteria infected diabetic wounds via photoswitchable cascade reaction
Source: Nat Commun. 2025 Oct 16;16:9198. doi: 10.1038/s41467-025-64242-z (PMC12533187; doi:10.1038/s41467-025-64242-z)
Supplement: Supplementary file 2 — Reporting Summary [file 41467_2025_64242_MOESM2_ESM.pdf]

nature portfolio

Corresponding author(s): Peng Li, Yuezhou Zhang

Last updated by author(s): Aug 10, 2025

Reporting Summary

Nature Portfolio wishes to improve the reproducibility of the work that we publish. This form provides structure for consistency and transparency in reporting. For further information on Nature Portfolio policies, see our Editorial Policies and the Editorial Policy Checklist.

Statistics

For all statistical analyses, confirm that the following items are present in the figure legend, table legend, main text, or Methods section.

n/a	Confirmed	
☐	☒	The exact sample size (*n*) for each experimental group/condition, given as a discrete number and unit of measurement
☐	☒	A statement on whether measurements were taken from distinct samples or whether the same sample was measured repeatedly
☐	☒	The statistical test(s) used AND whether they are one- or two-sided<br>*Only common tests should be described solely by name; describe more complex techniques in the Methods section.*
☒	☐	A description of all covariates tested
☒	☐	A description of any assumptions or corrections, such as tests of normality and adjustment for multiple comparisons
☐	☒	A full description of the statistical parameters including central tendency (e.g. means) or other basic estimates (e.g. regression coefficient) AND variation (e.g. standard deviation) or associated estimates of uncertainty (e.g. confidence intervals)
☐	☒	For null hypothesis testing, the test statistic (e.g. *F*, *t*, *r*) with confidence intervals, effect sizes, degrees of freedom and *P* value noted<br>*Give P values as exact values whenever suitable.*
☒	☐	For Bayesian analysis, information on the choice of priors and Markov chain Monte Carlo settings
☒	☐	For hierarchical and complex designs, identification of the appropriate level for tests and full reporting of outcomes
☒	☐	Estimates of effect sizes (e.g. Cohen's *d*, Pearson's *r*), indicating how they were calculated

Our web collection on statistics for biologists contains articles on many of the points above.

Software and code

Policy information about availability of computer code

Data collection	HPLC: ThermoFishe ICS5000; SEM: ZEISS Gemini SEM 300; TEM: Hitachi, HT7800; FTIR: Bruker, Tensor II; Raman: Renishaw inVia Raman Microscope; DR UV-vis: Shimadzu, UV-3600 Plus; EPR: Bruker EMXPLUS10/12; immunofluorescence: Thermo Fisher Scientific, EVOS FL Auto 2;
Data analysis	GraphPad Prism8 was used to perform general statistical analvses;<br>The following software was used for data analysis: ImageJ; Origin2021; XPSpeak41; MDI jade 6;

For manuscripts utilizing custom algorithms or software that are central to the research but not yet described in published literature, software must be made available to editors and reviewers. We strongly encourage code deposition in a community repository (e.g. GitHub). See the Nature Portfolio guidelines for submitting code & software for further information.

Data

Policy information about availability of data

All manuscripts must include a data availability statement. This statement should provide the following information, where applicable:
- Accession codes, unique identifiers, or web links for publicly available datasets
- A description of any restrictions on data availability
- For clinical datasets or third party data, please ensure that the statement adheres to our policy

All data underlying this study are available from the corresponding author upon request. Source data of Figures 2a-f, 3a-e, 4a-e, 6b, 6d, 7b-c, 7e, and 8e, and Supplementary Figures S3-S5, S7-S10, S12-S17, S19, S22, and S26-S30 are provided in a Source Data file.

Research involving human participants, their data, or biological material

Policy information about studies with human participants or human data. See also policy information about sex, gender (identity/presentation), and sexual orientation and race, ethnicity and racism.

Reporting on sex and gender	N/A
Reporting on race, ethnicity, or other socially relevant groupings	N/A
Population characteristics	N/A
Recruitment	N/A
Ethics oversight	N/A

Note that full information on the approval of the study protocol must also be provided in the manuscript.

Field-specific reporting

Please select the one below that is the best fit for your research. If you are not sure, read the appropriate sections before making your selection.

☒ Life sciences ☐ Behavioural & social sciences ☐ Ecological, evolutionary & environmental sciences

For a reference copy of the document with all sections, see nature.com/documents/nr-reporting-summary-flat.pdf

Life sciences study design

All studies must disclose on these points even when the disclosure is negative.

Sample size	No statistical methods were used to predetermine sample size. For in vivo studies with animals, sample sizes were determined by our preliminary experiments.<br>For in vitro studies, the sample size of each experiment was indicated in the figure legends.
Data exclusions	No data were excluded.
Replication	All experiments were conducted at least three independent times with similar results as indicated in the figure legends. All replicates were successful.
Randomization	Cells, organoids and mice were randomly allocated to either experimental or control groups.
Blinding	Histopathological evaluation of skin tissues was performed independently by two pathologists. All other data were based on quantitative analysis of phenotypes, and blinding was not required.

Reporting for specific materials, systems and methods

We require information from authors about some types of materials, experimental systems and methods used in many studies. Here, indicate whether each material, system or method listed is relevant to your study. If you are not sure if a list item applies to your research, read the appropriate section before selecting a response.

Materials & experimental systems

n/a	Involved in the study	
☒	☐	Antibodies
☐	☒	Eukaryotic cell lines
☒	☐	Palaeontology and archaeology
☐	☒	Animals and other organisms
☒	☐	Clinical data
☒	☐	Dual use research of concern
☒	☐	Plants

Methods

n/a	Involved in the study	
☒	☐	ChIP-seq
☒	☐	Flow cytometry
☒	☐	MRI-based neuroimaging

Eukaryotic cell lines

Policy information about cell lines and Sex and Gender in Research

Cell line source(s)	The cell lines NIH/3T3 and RAW 264.7 murine macrophage-like were obtained from Wuhan Servicebio Technology Co., Ltd.<br>The cell lines HUVEC was obtained from Procell Life Science & Technology Co., Ltd.

Authentication	Al cell lines were authenticated using STR analysis according to the lCLAC guidelines.
Mycoplasma contamination	All cell lines were routinely tested for mycoplasma and tested negative.
Commonly misidentified lines (See ICLAC register)	No commonly misidentified cell lines were used in this study.

Animals and other research organisms

Policy information about studies involving animals; ARRIVE guidelines recommended for reporting animal research, and Sex and Gender in Research

Laboratory animals	The mice were used at 6-8 weeks of age.
Wild animals	The study did not involve wild animals.
Reporting on sex	All experimental mice were male.
Field-collected samples	The study did not involve samples collected from the field.
Ethics oversight	All animal-based experimental studies were conducted in accordance with the guidelines of the Administration of Laboratory Animals of China and approved by the animal ethics committee of Northwestern Polytechnical University (Number: NPU201906).

Note that full information on the approval of the study protocol must also be provided in the manuscript.

Plants

Seed stocks	None
Novel plant genotypes	None
Authentication	None
